# Effect of different pre-analytical conditions on plasma lactate concentration

**DOI:** 10.11613/BM.2018.020701

**Published:** 2018-04-15

**Authors:** Ivana Rako, Ana Mlinaric, Monika Dozelencic, Gordana Fressl Juros, Dunja Rogic

**Affiliations:** 1 Department of Laboratory Diagnostics, University Hospital Centre Zagreb, Croatia; 2 Department of Biochemistry and Haematology, General Hospital “Dr. Ivo Pedišić”, Sisak, Croatia; 3 Department of Clinical Laboratory Diagnostics, Children’s Hospital Srebrnjak, Zagreb, Croatia

**Keywords:** plasma lactate, blood analysis, blood specimen collection, haemolysis, pre-analytical phase

## Abstract

**Introduction:**

Plasma lactate is a frequently used and important parameter for medical decision making. To setup a pre-analytical algorithm, we aimed to investigate the influence of different test tube additives, aliquoting, ice storage and haemolysis on plasma lactate concentrations for possible sparing critically ill (ICU) patients of additional blood drawing.

**Materials and methods:**

In our study (N = 177), lactate concentration and haemolysis index (HI) were measured in aliquoted (AHP) and unaliquoted (HP) Li-heparin, NaF/K_3_EDTA and NaF/KOX plasma, centrifuged within 15 minutes after venipuncture, on Cobas c501 analyzer. Differences were tested using the Wilcoxon’s test and Passing-Bablok regression. Clinical accuracy of results was assessed in 107 ICU patients based on reference interval and clinical decision limits.

**Results:**

Lactate concentrations did not differ in NaF/K_3_EDTA and NaF/KOX plasma (P = 0.855). No clinically significant difference of AHP compared to NaF/K_3_EDTA lactate was found (y = 0.13 (0.08 to 0.19) + 1.02 (0.99 to 1.08) x) if samples were aliquoted within 30 minutes after venipuncture. On contrary, lactate concentrations in HP showed significant proportional difference (y = 0.07 (- 0.12 to 1.24) + 1.37 (1.22 to 1.56) x) and were clinically incorrect in 14% of patients. Transport in ice bath increases HI in NaF/K_3_EDTA (P < 0.001), but without influencing lactate results compared to room temperature (y = 0.03 (- 0.06 to 1.00) + 1.05 (0.99 to 1.11) x).

**Conclusions:**

Lactate determination in HP is unacceptable because of high proportional error and high risk of clinical inaccuracy compared to NaF/K_3_EDTA. If pre-analytical conditions are met, AHP, NaF/K_3_EDTA and NaF/KOX plasma can be used interchangeably. Aliquoted Li-heparin samples alow measurement of other biochemical tests from a single tube and can spare ICU patients from additional blood drawing. Storage in ice bath provides no additional stabilization in NaF/K_3_EDTA tubes.

## Introduction

Blood lactate is a commonly ordered blood test for critically ill patients in intensive care units (ICU) with high risk of multiple organ failure following cardiogenic, haemorrhagic or septic shock and acute pulmonary insufficiency (*1*). Increased blood lactate is an early warning indicator of impaired tissue perfusion and oxygenation which makes lactate an important urgent test for directly assessing the shock severity and mortality rates (*2*). Acute changes in lactate concentrations can influence clinical management and application of therapy, so lactate measurements might be performed from 2 - 3 times *per* day to every 1 - 2 hours in acute conditions (*2*). Decreasing or persistently low concentrations of blood lactate during critical illness signify a favourable prognosis (*3*). However, frequent blood sampling and copious amounts of blood drawn represent a serious problem due to the phlebotomy related blood loss in ICU patients (*4*).

According to the Clinical and Laboratory Standards Institute (CLSI) guidelines, in order to obtain rapid results, it is recommended to use biosensor-based electrochemical whole blood lactate measurements at the point-of-care testing system (POCT), utilizing capillary or arterial whole blood drawn in a heparinized capillary tube or syringe (*5*). However, in our country many hospitals still use plasma for lactate determination in a central laboratory (*6*). In this setting it is imperative to define the pre-analytical factors that affect the reliability of plasma lactate.

The most critical factors that influence lactate results are stabilizing additives (antiglycolytic agents), temperature and time of storage. Sodium fluoride (NaF) is the most common used stabilizer to prevent changes in lactate concentration (*7*). NaF inhibits the glycolytic enzyme enolase and is usually used in combination with an anticoagulant such as potassium oxalate (KOX), ethylenediaminetetraacetic acid (EDTA), lithium heparin (Li-heparin) or citrate (*8*). KOX has an additional inhibitory effect by inhibiting pyruvate kinase (*9*). For lactate determination, the reagent manufacturers recommend NaF/KOX or NaF/Na-heparin plasma (*10*). Although different combinations of stabilizing additives are offered by manufacturers, their mutual comparison is unknown.

Other factors that influence accurate lactate determination are temperature and storage. Clinical and Laboratory Standards Institute guidelines recommend placing the drawn blood sample in an ice bath immediately after stasis-free venepuncture, and centrifugation within 15 minutes (*11*). However, it was previously shown that storage on ice provides no additional stabilization compared to room temperature when blood is obtained correctly in tubes containing NaF/KOX (*7*). Li-heparin plasma is also acceptable, but blood must be taken on ice and plasma separated from cells within 15 minutes of collection (*10*). Additionally, placing the blood in an ice bath immediately after venepuncture can cause haemolysis which might affect lactate results and other biochemical tests in plasma. Due to the activity of glycolytic enzymes together with a certain amount of lactate released from ruptured erythrocytes, haemolysis may also have an impact on final lactate concentration by increasing lactate concentrations which opposes the desired ice-cooling effect (*12*). Although manufacturers of lactate assays declare interference of haemolysis index, the biological interference of haemolysis on plasma lactate is unclear.

The aim of our study was to assess the key pre-analytical elements which affect the reliability of obtained plasma lactate concentration. Effects of different stabilizing additives, aliquoting, ice storage and haemolysis were evaluated in three separate comparison studies with specific goals: I) to establish if we can use test tubes with different additives for lactate determination by comparing plasma lactate in NaF/K_3_EDTA and NaF/KOX tubes, initially and after 24 hours storage; II) to compare lactate concentration in NaF/K_3_EDTA tubes transported at room temperature with concentrations in the same tubes stored in ice bath and to investigate the influence of resulting haemolysis on plasma lactate concentrations; III) to assess if ICU patients can be spared an extra blood draw for lactate determination by comparing lactate concentration in Li-heparin plasma (HP) also routinely used for other urgent biochemical tests, Li-heparin plasma aliquot (AHP) and tube with stabilizer NaF/K_3_EDTA.

## Materials and methods

### Study design

This is prospective experimental study which includes three separate comparison studies: I) comparison of lactate in NaF/K_3_EDTA and NaF/KOX tubes; II) comparison of lactate concentration in NaF/K_3_EDTA tubes transported ice bath and at room temperature; and III) comparison of lactate values in HP, AHP and NaF/K_3_EDTA plasma. The study was performed on samples collected from March 2015 to March 2016, at University Hospital Centre Zagreb.

The study was performed according to the requirements of the Declaration of Helsinki, following the approval of our institution’s ethics committee. Informed consent was obtained from all participants.

### Subjects

A total number of subjects in all comparison studies was 177 (122 males and 55 females; median age 65, range 16 – 83 years).

Twenty participants (11 males and 9 females; median age 43, range 25 – 72 years) were included in comparison of lactate concentrations in NaF/K_3_EDTA and NaF/KOX test tubes. Seven were patients from the Department of Thoracic Surgery, and others were volunteers. We have included both patients and volunteers to reflect populations of two other comparison studies.

Pre-analytical stability of lactate in samples drawn in NaF/K_3_EDTA tubes was tested in 50 outpatients (26 males and 24 females; median age 61, range 16 – 83 years) by comparing the lactate concentration in the tube placed in ice bath directly after venipuncture and tube stored at room temperature before sample processing.

Comparison of lactate concentrations in HP, AHP and NaF/K_3_EDTA plasma were performed in samples of 107 patients (85 males and 22 females; median age 67, range 34 – 82 years) with suspected hypo perfusion or hypoxia from the Department of Thoracic Surgery Intensive Care.

### Blood sampling

For all samples, blood was collected with minimal stasis and centrifuged for 10 minutes at 2000 x g on a centrifuge Rotofix 32A (Hettich GmbH, Tuttlingen, Germany) within 15 minutes from venipuncture. Depending on the study design, tubes with NaF/K_3_EDTA (2mL), NaF/KOX (2mL) and Li-heparin (4mL) anticoagulants (Greiner Bio-One GmbH, Kremsmünster, Austria) were used.

For comparison of lactate in NaF/K_3_EDTA and NaF/KOX tubes, samples were collected in the morning hours after an overnight fast and sent to the laboratory at room temperature.

For comparison of lactate concentration in NaF/K_3_EDTA tubes transported at room temperature and in an ice bath, samples were collected in the morning hours after an overnight fast. To avoid haemolysis due to blood sampling, none of these tubes was the first sampling tube for outpatients.

For comparison of lactate in HP, AHP and NaF/K_3_EDTA plasma, samples were collected at any time of the day no matter the fasting state and sent to the laboratory at room temperature. Contrary to the CLSI guidelines we omitted storing Li-heparin and NaF/K_3_EDTA tubes in an ice bath to avoid possible haemolysis as HP sample was also used for other urgent biochemistry tests (*11*). This study also served for evaluation of possible sparing of excess blood draw for ICU patients. Li-heparin plasma was aliquoted immediately after centrifugation.

### Methods

All lactate measurements were carried out on Cobas c501 analyser (Roche Diagnostics GmbH, Mannheim, Germany) using the commercially available reagent Lactate Gen.2 (Roche Diagnostics GmbH, Mannheim, Germany). The manufacturer of the Lactate Gen.2 assay validated its performance on the Cobas c501 analyser with those determined using the same reagent on a Roche/Hitachi 917 analyser. A short verification of the method performance was performed in our laboratory before the study was conducted, according to HRN EN ISO 15189:2012 Medical laboratories - Requirements for quality and competence. During the 1-year study period, plasma lactate assay precision (CV) and accuracy (BIAS) were less than 2% and sigma value > 6. Quality control was performed every 12 hours using normal and high concentrations of commercially available control material (PreciControl ClinChem Multi 1 and PreciControl ClinChem Multi 2, Roche Diagnostics GmbH, Mannheim, Germany).

Haemolysis index (HI) was determined and quantified on Cobas c501 analyser using the commercially available reagent Serum Index Gen.2 (Roche Diagnostics GmbH, Mannheim, Germany). Haemolysis index of 10 corresponds to a haemoglobin concentration of 0.1 g/L. Manufacturer of lactate assay declares no significant interference up to HI of 1000.

For comparison of lactate in NaF/K_3_EDTA and NaF/KOX tubes, after blood sampling and centrifugation, NaF/K_3_EDTA and NaF/KOX primary tubes were immediately placed on the analyser and analysis was completed in about 1 hour after the draw time. Following the initial measurement, the samples were stored for 24 hours at 2 - 8 °C. Afterwards, the lactate measurement was repeated to compare the antiglycolytic effect of tube additives.

For comparison of lactate in NaF/K_3_EDTA tubes transported at room temperature and in ice bath samples, NaF/K_3_EDTA primary tubes were used for lactate and HI measurement and results were obtained within 1 hour from venipuncture.

For comparison of lactate concentrations in HP, AHP and NaF/K_3_EDTA plasma, Li-heparin tubes were aliquoted immediately after centrifugation and lactate was measured in AHP and HP at the same time with NaF/K_3_EDTA primary tube. Results were obtained within 1 hour from venipuncture at the same time for three parallel samples.

### Statistical analysis

Data were tested for normality using the Kolmogorov-Smirnov test. Statistical differences in data that did not follow normal distribution, and lower sample size data (N < 30), were tested using the Wilcoxon’s rank sum test for paired samples. Normally distributed data were tested for statistical differences with paired samples t-test. The comparability of results was tested using Passing-Bablok regression analysis and Bland-Altman analysis. Within-subject biological variation for lactate is 27% (*13*). Therefore differences in compared tubes higher than 27% were considered clinically significant. Pearson correlation was used to test the association of delta HI (HI in ice bath sample – HI in room temperature sample) and delta NaF/K_3_EDTA lactate (lactate in an ice bath sample – lactate at room temperature sample) between tubes transported at different temperatures. A P value < 0.05 was set as a level of statistical significance. Statistical analysis was performed using MedCalc Software v. 9.3.2.0. (Ostendt, Belgium). The clinical accuracy of lactate measured in HP and AHP compared to NaF/K_3_EDTA concentrations was assessed based on reference interval and clinical decision limits. Values were considered inaccurate if lactate in the compared tube (HP, AHP) was above the upper limit of reference interval (2.2 mmol/L), unlike the NaF/K_3_EDTA lactate. If the concentration of lactate increases beyond 3 – 4 mmol/L, there is an increased risk of associated acidosis and the possible need for therapy (*14*). At our institution, the concentration of 3 mmol/L is used as the limit of clinical decision-making. Therefore, if the lactate concentration in the compared tube was > 3 mmol/L, while the reference NaF/K_3_EDTA plasma lactate concentrations were < 3 mmol/L or within the reference interval, this measurement was considered highly inaccurate as the decisions based on this value could potentially cause patient mistreatment.

## Results

The measured lactate concentrations in NaF/K_3_EDTA and NaF/KOX tubes ranged from 0.5 to 2.3 mmol/L. Median and interquartile range (IQR) for different tubes, both initially and after 24 hours, are shown in Table 1. No statistical difference in lactate results or measured lactate concentration between NaF/K_3_EDTA and NaF/KOX tubes, both, in initial sample (P = 0.855) and 24 hours (P = 0.626) was found.

**Table 1:** Lactate results in different pre-analytical conditions

**STUDY 1 (N = 20)**								
		**NaF/K_3_EDTA**			**NaF/KOX**		P*	
Initial lactate concentration (mmol/L)		1.1 (0.7 - 1.6)			1.1 (0.7 - 1.5)		0.855	
Lactate after 24 h (mmol/L)		1.2 (1.0 - 1.9)			1.3 (1.0 - 1.8)		0.626	
**STUDY 2 (N = 50)**								
		**NaF/K_3_EDTA** **(ice bath)**			**NaF/K_3_EDTA (room temperature)**			**P^†^**
Lactate (mmol/L)		1.2 (± 0.49)			1.3 (± 0.51)			< 0.001
Haemolysis index		25 ± 12			16 ± 8			< 0.001
**STUDY 3 (N = 107)**								
	**NaF/K_3_EDTA**		**AHP**	P* (NaF/K_3_EDTA *vs.* AHP)		**HP**		P* (NaF/K_3_EDTA ***vs.* HP)**

Lactate (mmol/L)	1.0 (0.8 - 1.3)		1.1 (1.0 - 1.5)	< 0.001		1.6 (1.3 - 2.0)		< 0.001
Study 1 - comparison of lactate in NaF/K_3_EDTA and NaF/KOX tubes at baseline and after 24 h of storage. Study 2 - comparison of lactate concentration and haemolysis index in NaF/K_3_EDTA tubes at different transport temperature conditions. Study 3 - comparison of lactate values in HP, AHP and NaF/K_3_EDTA plasma. Data are presented as a median and interquartile range (IQR) or mean and standard deviation (SD) depending on sample size and the normality of distribution. P < 0.05 was considered statistically significant. AHP - aliquoted heparin plasma. HP - heparin plasma. *Wilcoxon’s rank sum test. ^†^Paired samples t-test.								

**Figure 1:**
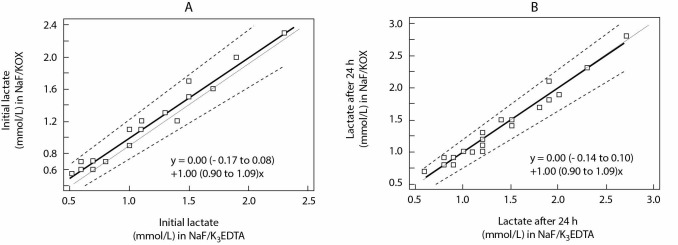
Comparison of lactate measured in NaF/K_3_EDTA and NaF/KOX plasma using Passing-Bablok regression analysis. (A) Initial lactate concentrations. (B) Lactate concentrations after 24 hour storage. The regression line equation (Y = A (95% CI) + B (95% CI) x) is shown in the box. 95% CI – confidence intervals of 95%. A - regression line’s intercept. B - regression line’s slope. Solid line - regression line. Dashed lines - 95% CI for the regression line. Dotted line - identity line (x = y).

**Figure 2:**
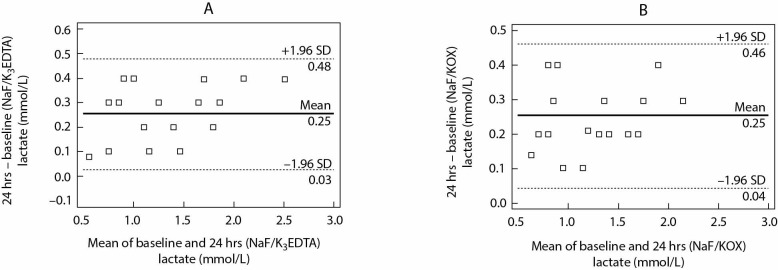
Comparison of lactate measured in (A) NaF/K_3_EDTA and (B) NaF/KOX plasma using the Bland-Altman analysis. Solid line (mean) – mean difference. Dashed lines (SD) – standard deviation. A mean difference of 0.25 mmol/L in lactate concentration after 24h storage in both tubes was found.

Passing-Bablok regression analysis showed no proportional nor constant error in determining lactate concentration between the two tubes with different anticoagulants, both in initial measurement and after 24 hours (Figure 1). Mean difference in lactate concentration after 24 hours was 0.25 mmol/L for both test tube types (Figure 2).

**Figure 3:**
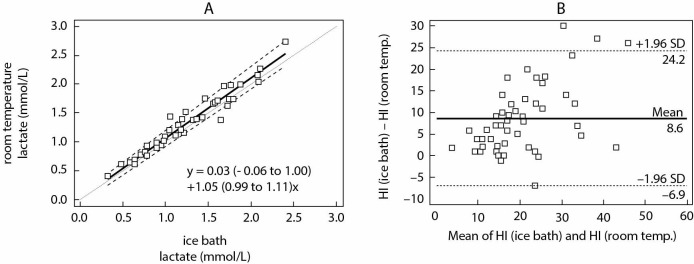
(A) Passing-Bablok regression analysis showing the comparison of lactate measured in NaF/K_3_EDTA tubes put in ice water bath compared to tubes kept at room temperature. The regression line equation (Y = A (95% CI) + B (95% CI) x) is shown in the box. 95% CI – confidence intervals of 95%. A - regression line’s intercept. B - regression line’s slope. Solid line - regression line. Dashed lines - 95% CI for the regression line. Dotted line - identity line (x = y). (B) Bland-Altman analysis showing the comparison of haemolysis index (HI) measured in ice bath and room temperature conditions. Solid line (mean) – mean difference. Dashed lines (SD) – standard deviation. A mean difference of 9 in samples that were put in ice water bath after venipuncture compared to samples that were kept at room temperature was found.

Lactate concentrations measured in NaF/K_3_EDTA tubes placed in ice bath were significantly lower than those measured in tubes stored at room temperature (P < 0.001). Mean value and standard deviation (SD) for plasma lactate results and HI obtained at different storage temperature is shown in Table 1. However, there was no proportional nor constant error obtained by Passing-Bablok analysis between the two tested temperature transport conditions. Haemolysis index was higher in tubes that were transported in an ice bath (HI mean difference 9, P < 0.001) (Figure 3). We did not find any correlation between delta HI and delta lactate concentration in tubes transported at room temperature and an ice bath (r = 0.20; P = 0.156).

**Figure 4:**
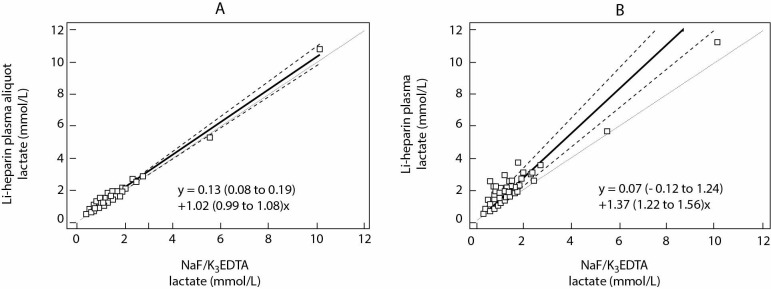
Comparison of results using Passing-Bablok regression analysis. (A) Li-heparin plasma aliquot and NaF/K_3_EDTA and (B) heparin plasma and NaF/K_3_EDTA. The regression line equation (Y = A (95% CI) + B (95% CI) x) is shown in the box. 95% CI – confidence intervals of 95%. A - regression line’s intercept. B - regression line’s slope. Solid line - regression line. Dashed lines - 95% CI for the regression line. Dotted line - identity line (x = y).

Lactate concentrations measured in AHP, HP and NaF/K_3_EDTA were between 0.3 and 11.3 mmol/L. The results are shown in Table 1. Wilcoxon’s rank sum test results showed a statistically significant difference in the concentration of lactate measured in AHP and HP compared to NaF/K_3_EDTA (P < 0.001). Passing-Bablok regression analysis of values in AHP compared to NaF/K_3_EDTA showed a constant error of 0.13 mmol/L in lactate concentration. Also, there was a high proportional error of 1.37 between HP compared to NaF/K_3_EDTA (Figure 4). HP lactate concentrations were clinically inaccurate in 14% of patients. All lactate concentrations measured in AHP were accurate based on clinical criteria (Table 2).

**Table 2:** Clinical accuracy of lactate measured in HP and AHP compared to NaF/K_3_EDTA plasma

	**Lactate (mmol/L)**	**NaF / K_3_EDTA**		
	**0.5 – 2.20**	**2.21 – 3.0**	**> 3.01**
**HP**	**0.5 – 2.20**	89	0	0
	**2.21 – 3.0**	10	1	0
	**> 3.01**	2	3	2
**AHP**	**0.5 – 2.20**	101	0	0
	**2.21 – 3.0**	0	4	0
	**> 3.01**	0	0	2
The clinical accuracy was assessed based on reference interval (0.5 - 2.2 mmol/L) and clinical decision limits (> 3 mmol/L). Values were considered inaccurate if lactate in the compared tube (HP, AHP) was above the upper limit of reference interval or > 3 mmol/L, unlike the NaF/K_3_EDTA lactate. AHP - aliquoted heparin plasma. HP - heparin plasma.				

## Discussion

In this study, we investigated the influence of different test tube additives, aliquoting, ice storage and haemolysis on plasma lactate concentrations through three different comparison studies.

Although manufacturers offer different test tubes and additives for plasma lactate determination, NaF/K_3_EDTA tube is not recommended by the manufacturer of the lactate assay. We showed that lactate concentration in NaF/K_3_EDTA is comparable to the recommended NaF/KOX plasma. To the best of our knowledge, we were unable to find any previous comparison studies with recommended tube. It was previously shown that lactate is stable in NaF/KOX at least 30 min at room temperature before centrifugation with the increase of only 0.2 mmol/L during the first hour at room temperature (*7*). Interestingly, we also observed no difference in lactate in NaF/KOX and NaF/K_3_EDTA tubes even after 24 hours of storage. These results confirm the previously reported stability of lactate collected in the tube with NaF stabilizer and stored at + 4 °C (*15*). These combinations of stabilizing additives have an equal antiglycolytic effect and very strongly inhibit lactate production *in vitro* for an extended period.

In the second study, we compared lactate concentration in NaF/K_3_EDTA tubes transported at room temperature with those in an ice bath. We confirmed that either way of temperature processing would not lead to a clinically different result. Our results are in accordance with the previous studies that showed that separating plasma from cells and its storage in ice bath are not necessary for lactate stability when blood is drawn with the stabilizer (*7*, *16*). Haemolysis can influence lactate results in the plasma stabilized with NaF/KOX, but there are no findings about correlation between HI and the lactate concentration (*17*). Our results showed that samples transported in ice bath had statistically higher haemolysis index compared to those transported at room temperature. However, difference in haemolysis does not correlate with the differences in lactate concentrations obtained at different transport temperatures. The impact of haemolysis index on plasma lactate concentration is highly individual and depends on a number of factors that can influence lactate metabolism *in vitro*: temperature, time, oxygen content in the sample, content of haemoglobin and the number of blood cells which act as reservoirs of glycolytic enzymes that increase lactate during sample storage (*1*, *18*).

Unlike the measurement of plasma lactate on biochemistry analyser, point-of-care testing (POCT) of whole blood lactate is the more pragmatic approach and increasingly justified based on medical benefits (*19*). However, many hospitals still use plasma sample because POCT is not established or financially feasible, especially for frequent lactate determination. Furthermore, in some cases, colorimetric measurements of lactate in arterial or venous plasma can perform better than potentiometry in whole blood because of less analytical interferences (*9*, *20*-*22*). Despite this, studies on pre-analytical effects on plasma lactate measurement are scarce.

For ICU patients it is necessary to perform complete laboratory testing several times a day for estimating patient clinical status (*23*). The organ dysfunction can be assessed using different scoring systems that quantify abnormalities according to clinical findings, laboratory data or therapeutic interventions. The predominantly current use scoring system is SOFA (Sequential Organ Failure Assessment). Laboratory tests as creatinine and bilirubin measured in heparin plasma are needed for score calculation. Additionally, lactate measurements along with other urgent laboratory tests measured in the heparin plasma can help identify patients at risk (*24*). Guidelines recommend that lactate should either be measured in the whole blood by the POCT instrument or it is necessary to draw an additional tube with stabilizer for plasma testing (*5*, *11*). For this reason, we wanted to assess if ICU patients can be spared an extra blood draw for lactate determination by comparing lactate in Li-heparin plasma also routinely used for other urgent biochemical tests. For HP, the observed proportional error of 37% is unacceptable considering the established 27% within-subject biological variance for lactate (*13*). That error is particularly unacceptable given the narrow reference interval for plasma lactate. We also made a clinical accuracy assessment of lactate results obtained in AHP and HP compared to NaF/K_3_EDTA plasma. Alarmingly, risk analysis for HP showed a moderate to high risk of clinical error in a high percentage of patients (14%). Therefore not-aliquoted Li-heparin plasma transported at room temperature is unsuitable for lactate determination (*25*). On contrary, clinical accuracy analysis showed that same clinical decision would be made consistently based on the AHP lactate concentrations. Therefore, AHP can be used for lactate determination which gives the opportunity of determining lactate from the same tube as other urgent biochemical tests and thus sparing ICU patients from an additional blood draw. To the best of our efforts, we were unable to find any similar study to compare our findings.

We acknowledge that the current study has some potential limitations. To spare the patients of large amount of blood sampling for study purposes, our hypotheses were tested on three separate patient groups of different sample size. Furthermore, the study that compared NaF/K_3_EDTA and NaF/KOX lactate concentrations included only 20 patients. Also, the majority of patients have initial lactate concentrations in the normal range and entire concentration range is not equally represented. However, this study elucidates some dilemmas about the pre-analytical phase of plasma lactate determination regarding different additives, storage in an ice bath and resulting haemolysis because no similar study has been reported. Additionally, results of this study show the possibility to reduce blood sampling in ICU patients when plasma lactate is ordered with other urgent biochemical tests. Also, these results contribute to the development of the appropriate guidelines to be applied in clinical practice in the pre-analytical phase of plasma lactate determination.

We conclude that AHP, NaF/K_3_EDTA and NaF/KOX plasma can be used interchangeably for lactate determination under the certain pre-analytical conditions. It is not necessary to put test tubes in ice bath directly after venipuncture if blood is drawn with NaF/K_3_EDTA stabilizer and centrifuged within 15 minutes after venipuncture. If Li-heparin test tubes are transported at room temperature, plasma must be aliquoted immediately after centrifugation. This algorithm avoids potential *in vitro* haemolysis due to ice-bath temperature conditions and spares ICU patients from excessive blood sampling by allowing the measurement of all urgent biochemical tests including lactate from a single Li-heparin tube. Lactate measurement in unaliquoted HP is unacceptable because of high proportional error and high risk of clinical inaccuracy in comparison to NaF/K_3_EDTA tubes.
